# Therapeutic Evaluation of the Suprachoroidal Space for Stem Cell Delivery in Retinal Degeneration

**DOI:** 10.1155/sci/1374159

**Published:** 2026-04-27

**Authors:** Hyun Hee Seo, Eun Ju Lee, Yoon Young Kim, Seung-Yup Ku, Chang Ki Yoon

**Affiliations:** ^1^ Interdisciplinary Program in Stem Cell Biology, Seoul National University College of Medicine, Seoul, 03080, Republic of Korea, snu.ac.kr; ^2^ Biomedical Research Institute, Seoul National University Hospital, Seoul, 03080, Republic of Korea, snuh.org; ^3^ Institute of Reproductive Medicine and Population, Medical Research Center, Seoul National University, Seoul, 03080, Republic of Korea, snu.ac.kr; ^4^ Department of Obstetrics and Gynecology, Seoul National University Hospital, Seoul, 03080, Republic of Korea, snuh.org; ^5^ Department of Ophthalmology, Seoul National University Hospital, Seoul, 03080, Republic of Korea, snuh.org

**Keywords:** human embryonic stem cell-derived mesenchymal stem cells, intravitreal injections, retinal degeneration, stem cell delivery, suprachoroidal injections, suprachoroidal space

## Abstract

The route of administration is a crucial determinant of the success of stem cell‐based therapies, especially in intraocular applications. It significantly influences therapeutic outcomes and the risk of adverse events. We aimed to evaluate the utility of the suprachoroidal space (SCS) as a delivery route for human embryonic stem cell‐derived mesenchymal stem cells (hESC‐MSCs) in a rabbit model of retinal degeneration in comparison with the intravitreal (IVT) route. A custom‐designed, three‐dimensional‐printed needle cap was employed to ensure precise hESC‐MSC delivery into the SCS via a relatively less invasive approach. Green fluorescent protein (GFP)‐induced hESC‐MSC expression was monitored for 7 weeks to assess persistence and localization. Therapeutic potential was assessed using electroretinography (ERG) as well as histological and immunofluorescence examinations on Day 28. Flourscence signals were still detectable up to Week 2, which indicated precise delivery to the posterior segment of the eye. There were significant ERG improvements in the hESC‐MSC‐treated group compared with those in the phosphate‐buffered saline‐treated group. Furthermore, the hESC‐MSC‐treated group showed a significant increase in outer nuclear layer thickness and cell number, as well as upregulated rhodopsin and opsin expression. Additionally, there was minimal inflammation in SC‐injected eyes compared with that in IVT‐injected eyes on Day 14. Our findings highlight the potential of the SCS as an optimal route for hESC‐MSC delivery. Specifically, it allows retinal targeting that significantly enhances visual function restoration while minimizing adverse events.

## 1. Introduction

Stem cell‐based therapies have emerged as a promising approach for restoring vision loss due to irreversible retinal diseases [1]. However, several challenges persist, including concerns regarding efficacy, safety, dose optimization, quality control, and the reliable supply of therapeutic cells [2]. A critical challenge is the route of administration, which plays a key role in clinical applications. Intraocular delivery can significantly impact the treatment efficacy and duration, inflammation and immune responses, and risk of adverse events [2–5].

Initially, stem cell‐based therapies focused on replacing damaged retinal cells using human embryonic stem cell (hESC)‐derived or induced pluripotent stem cell‐derived retinal pigment epithelial (RPE) cells from allogeneic and autologous sources. In cell replacement therapy, subretinal injections are commonly used for stem cell delivery. This approach, which allows precise delivery of cells to the degeneration site between the retina and RPE, has demonstrated significant therapeutic outcomes. However, it is technically demanding and requires invasive procedures, including pars plana vitrectomy or retinotomy. Although postoperative complications are generally manageable, they may include retinal detachment, subretinal hemorrhage, and vitreous hemorrhage [3, 4, 6]. Additionally, retinal atrophy at the subretinal bleb site was reported following the first approved retinal gene therapy [7]. Despite the relative immune privilege of the subretinal space, immunosuppressant use is often recommended, especially for older patients who have a risk of additional side effects [8–12].

Mesenchymal stem cells (MSCs) have garnered significant attention in regenerative medicine due to their immunomodulatory properties, therapeutic potential, and ease of procurement [2, 13, 14]. IVT injections have become a common delivery method for MSCs due to their widespread application in managing various ophthalmic diseases [15, 16]. Specifically, IVT injections have gained popularity following the introduction of antivascular endothelial growth factor therapies for age‐related macular degeneration [16]. Additionally, IVT injection is preferred given its procedural simplicity, minimally invasive nature, and low incidence of complications [17].

Several animal studies have investigated the therapeutic efficacy of IVT‐MSC delivery. Based on these studies, a primary concern is suboptimal cell distribution within the vitreous body, which may be attributed to their dispersion within the vitreous cavity and compromises targeted delivery to the posterior segment of the eye. Additionally, inflammatory responses associated with IVT‐MSC delivery further undermine the therapeutic efficacy [3, 4, 6, 15, 17–19]. For example, IVT injection of adeno‐associated viral (AAV) vectors in retinal gene therapy elicits the production of neutralizing antibodies, which highlights the limitations of this approach. Such humoral immune responses may compromise therapeutic outcomes and pose safety risks. To this point, a clinical trial on IVT gene therapy for diabetic macular edema was suspended due to the occurrence of inflammation and hypotony [9, 20, 21].

The suprachoroidal space (SCS), which is located between the sclera and choroid, is an emerging route for posterior segment drug delivery. The SCS becomes prominent under pathological conditions, including suprachoroidal (SC) fluid accumulation [22, 23]. In 2021, XIPERE (triamcinolone acetonide injectable suspension, Clearside Biomedical, USA) became the first therapy approved by the U.S. Food and Drug Administration (FDA) for use within the SCS, which specifically targets macular edema associated with uveitis. This milestone prompted further investigation of the utility of this route in delivering various therapeutic agents, with extensive investigation of the related pharmacokinetics and safety [24]. The SC route enables rapid and targeted delivery to the posterior segment of the eye, which allows swift diffusion of materials across the sclera without entering the vitreous body and thus minimizes the risk of side effects [15, 23, 25].

This injection procedure has demonstrated a robust safety profile with no significant adverse events [15, 26, 27]. Additionally, the PEACHTREE clinical trial revealed marked improvements in visual acuity, highlighting the therapeutic potential of the SC approach [28]. Accordingly, the SCS has been increasingly recognized as a transformative delivery route for stem cell‐based therapies, allowing precise targeting and minimal invasiveness. However, the persistence of administered cells, therapeutic efficacy, and potential intraocular inflammatory responses associated with MSCs delivered via this route remain unclear.

This study aimed to perform a comparative evaluation of the therapeutic effects of human embryonic stem cell‐derived mesenchymal stem cell (hESC‐MSC) delivery via the SC and IVT routes in a rabbit model of retinal degeneration (RD). We selected rabbits due to their anatomical and biochemical similarities to humans, which makes them an ideal model for drug delivery studies; moreover, their relatively large ocular dimensions compared with rodents have facilitated the assessment of a custom‐designed injector for SC administration [29]. Specifically, this study aimed to elucidate the potential advantages and limitations of the SC route for hESC‐MSCs delivery relative to the IVT approach in the context of RD.

## 2. Materials and Methods

### 2.1. Delivering to the SCS

**Figure 1:**
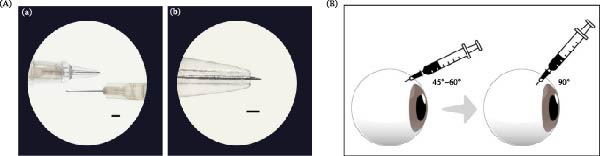
(A) Stereo microscope image of a three‐dimensional‐printed cap applied to a 30‐gauge ½ needle. (a) Stereo microscope image showing a 30‐gauge ½ needle. The left image shows the needle with a 3D‐printed cap attached, which limited the penetration depth to 920–950 µm for delivery into the suprachoroidal space (SCS). The right image shows the needle without the cap, revealing its full 13‐mm length. (b) A magnified view of the capped needle from (a), highlighting the bevel orientation. *Scale bar* = 1 mm. (B) Schematic representation of the suprachoroidal injection technique. The needle is initially positioned at a 45° angle, with the bevel facilitating scleral penetration. Upon scleral penetration, the needle is adjusted perpendicularly to the scleral surface. Subsequently, cells are slowly injected, with a 30‐s pause to prevent backflow and a brief stationary period to facilitate stable delivery. 3D, three‐dimensional.

Microneedles were utilized to deliver stem cells into the SCS with minimal risk of retinal damage. A custom‐designed needle cap was created using three‐dimensional (3D) printing technology. This cap was engineered to fit a 30‐gauge ½ needle (BD PrecisionGlide needle, NJ, USA), with the needle’s exposure being limited to ≤1 mm. This ensured that the syringe lumen could reach the SCS without penetrating the retinal layers (Figure 1A). The 3D‐printed cap, made of <1 mm transparent plastic, was designed to maintain a 45°–60° angle of contact with the scleral surface, with the needle bevel facing upward during the injection process. A 1‐mL Luer Lock syringe (BD, OH, USA) was used to prevent cell expulsion during aspiration. The overall injection process is illustrated in Figure 1B. The feasibility of the SC route was validated using a red polystyrene microsphere (Invitrogen 15 µm, FluoSpheres polystyrene microsphere, 580/605) in rabbit eyes with customized 3D‐printed needle caps. Representative cryosection images were obtained using a confocal microscope (Leica STELLARIS 8, Upright, DE, USA) (Figure S1).

### 2.2. Cell Preparation for the Injection

We obtained hESC‐MSCs (2.5 × 10^7^ cells per vial, passage 12; Dae Woong Pharmaceutical Co., Ltd., KK, Korea) for research purposes. Cryovials stored in liquid nitrogen (LN2) were quickly thawed in a 37°C water bath. Thawed cells were gently rinsed following the manufacturer’s instructions. Across all experiments, each injection comprised 3 × 10^6^ cells suspended in 100 µL of phosphate‐buffered saline (PBS), regardless of the delivery route, and was maintained on ice. For cell tracking, hESC‐MSCs were labeled with a green fluorescent protein (GFP) through transduction with GFP lentivirus (LugenSCI, Seoul, KR) following the manufacturer’s instructions, with modifications to optimize transduction efficiency. Successful labeling was verified using light microscopy and confirmed by brightfield and fluorescence microscopy (Nikon Eclipse Ci‐L, DE, USA) (Figure S2 A, B). Immunophenotypic characterization of GFP‐transduced hESC‐MSCs was further confirmed by flow cytometry (Figure S3).

### 2.3. In Vivo Experiment Procedure

All study protocols and procedures were approved by the Institutional Animal Care and Use Committee of Seoul National University Hospital (22‐0329‐S1A0, 23‐0361‐S1A1). New Zealand white rabbits, weighing 2–2.5 kg, were obtained from Koatech (Seoul, KR) and housed under a 12‐h light/dark cycle. Anesthesia was intramuscularly administered using a combination of ketamine (35 mg/kg, ketamine hydrochloride 50 mg/mL injection, Huons Co., Ltd., KK, KOR) and xylazine hydrochloride (5 mg/kg, Rompun, 0.2 mL/kg, Bayer Corp., Shawnee Mission, KS, USA).

To establish baseline values, electroretinogram (ERG) recording was performed before RD induction. RD was induced using sodium iodate (15 mg/kg body weight, Sigma–Aldrich Corp., St. Louis, MO, USA) to yield partial vision rather than bilateral blindness, as confirmed by ERG analysis. Sodium iodate (10 mg/mL in 0.9% saline) was injected into the marginal ear vein under anesthesia, and 24 hr after RD induction, cells or PBS was injected [30, 31]. The procedure was adapted from in‐house protocol (Figure S4 A–H). The rabbits exhibited normal locomotion and feeding behavior, with regular monitoring of food intake and overall health.

All injections were performed under systemic anesthesia induced using ketamine hydrochloride and xylazine hydrochloride, with topical proparacaine hydrochloride (ALCAINE, Alcon Laboratories, Inc., TX, USA) applied to the cornea, followed by sanitization with 2.5% povidone–iodine (Green Povidone Iodine Solution, Green Pharmaceutical Co., Ltd., KK, KOR). SC and IVT injections were administered 3 mm posterior to the limbus in the superonasal quadrant under microscopic guidance. The SC injection procedure is described in Section 2.1, while the IVT injection was carefully performed to minimize the risk of retinal injury.

At the end of the experiment, animals were euthanized by intravenous administration of potassium chloride (KCl) under deep anesthesia, in accordance with institutional and international guidelines.

#### 2.3.1. Time‐Dependent Persistence Using GFP‐Transduced hESC‐MSCs Following SC and IVT Injections

First, we examined the intraocular persistence and distribution of GFP‐transduced hESC‐MSCs following SC‐ and IVT injections. Bilateral eye injections of GFP‐labeled hESC‐MSCs were performed in seven healthy rabbits, with one eye undergoing SC delivery and the contralateral eye undergoing IVT delivery. On Days 1, 4, 7, and 10, as well as Weeks 2, 5, and 7, both eyes were enucleated, frozen in OCT compound, and cryosectioned perpendicular to the retina across the optic nerve at a 10‐µm thickness. Sections were washed with PBS, stained with 4′, 6‐diamidino‐2‐phenylindole (DAPI), and visualized using confocal microscopy.

#### 2.3.2. Evaluation of the Efficacy of SC‐Delivered hESC‐MSCs up to Day 28

Second, we evaluated the therapeutic efficacy of hESC‐MSCs delivered via the SC route over a 28‐day observation period. Here, seven RD rabbits were administered with hESC‐MSCs in one eye (SC‐delivered hESC‐MSCs) and PBS in the contralateral eye (SC‐delivered PBS). ERG recording was performed on baseline (pre‐RD induction), Day 0 (24 hr after RD induction; immediately before hESC‐MSCs/PBS injections), on Day 14, and Day 28 post‐injection. Both eyes were enucleated on Day 28 for histology and molecular analyses.

#### 2.3.3. Assessment of the Intraocular Inflammation Responses up to Day 14 Following SC and IVT Injections

Third, we investigated intraocular inflammation following hESC‐MSC administration via the SC and IVT routes. Here, four RD rabbits were used. One rabbit remained untreated. The other three rabbits received hESC‐MSC injections via the SC route in one eye (SC‐injected eye) and the IVT route in the other (IVT‐injected eye). On Day 14, all eyes were enucleated under anesthesia and assessed through histological analysis as well as immunofluorescence staining using CD45 and CD8 antibodies.

### 2.4. Electroretinography (ERG)

Retinal function was assessed using ERG following the International Society for Clinical Electrophysiology of Vision 2022 guidelines [32]. ERG recordings were obtained under dark‐ and light‐adapted conditions using full‐field stimulation (15 cd.s/m^2^) with a Celeris stimulator system (Diagnosys LLC, USA). Baseline measurements were obtained prior to RD induction. For ERG recording under dark‐adapted conditions, rabbits were kept in complete darkness for 40 min prior to recording. Next, the rabbits were anesthetized, and pupils were dilated using topical 5% phenylephrine hydrochloride followed by 5% tropicamide. The reference and ground electrodes were placed subcutaneously at the intraocular region and near the tail, respectively. The a‐ and b‐wave amplitudes were recorded with noise filtration set to 50–60 Hz. All procedures were performed in a dark room, with the animal’s body temperature being maintained at 37°C using a bottom heating pad to prevent hypothermia.

### 2.5. Histology and Immunofluorescence Analysis

The enucleated eyes were fixed in 10% formaldehyde for 1 week and processed using an automated tissue processor (Leica PELORIS II, MI, USA). Paraffin sections (4‐µm thickness) were cut through the injection site and optic nerve, followed by staining with hematoxylin and eosin using an automated stainer (Leica Autostainer XL, Leica Biosystems, Nussloch, Germany) to assess morphological changes. For immunofluorescence analysis, deparaffinized sections were rehydrated, subjected to antigen retrieval in preheated 1x citrate buffer, and permeabilized with 0.5% Triton X‐100. Sections were blocked in blocking buffer for 1 hr, followed by incubation overnight at 4°C with primary antibodies: antirhodopsin (1:250: mouse, Sigma–Aldrich), anti‐opsin (1:500; mouse, Sigma–Aldrich), CD45 (1:500; rabbit, Invitrogen, MA, USA), and CD8 (1:250; mouse, Novus Biologicals). The next day, the sections were incubated with Alexa Fluor‐conjugated secondary antibodies (Invitrogen) at room temperature and counterstained with DAPI (Fluoroshield Mounting Medium with DAPI, Abcam, Cambridge, UK). Confocal imaging was performed using a Leica STELLARIS 8 microscope (Upright, DE, USA).

### 2.6. Outer Nuclear Layer (ONL) and Inner Nuclear Layer (INL) Thickness Analysis

ONL and INL thicknesses were quantified using ImageScope software (Aperio, Leica Biosystems, USA) on hematoxylin and eosin‐stained images of whole sagittal sections. Images were visualized at 100 magnifications; further, thickness was manually measured at three randomly selected points within 2.0–2.5 mm from the optic nerve head. The average thickness was calculated for each sample. Among the seven samples, we selected five with stable imaging quality for analysis. Thickness measurements were bilaterally performed in both the nasal and temporal regions [33, 34]. For cell counting analysis, DAPI‐stained images were obtained from a defined nasal region that corresponded to the area used for immunofluorescence imaging. The number of nuclei in the ONL was manually quantified using ImageJ software (NIH, USA).

### 2.7. Statistical Analysis

All quantitative data were recorded using Microsoft Office Excel 2017. Statistical analyses were performed using GraphPad Prism (Version 10 for Windows). We employed one‐way and two‐way analysis of variance or Mann–Whitney *U* tests. Results are presented as the mean ± standard error. Statistical significance is indicated using asterisks:  ^∗^ *p* < 0.05,  ^∗∗^ *p* < 0.01,  ^∗∗∗^ *p* < 0.001, and  ^∗∗∗∗^ *p* < 0.0001.

## 3. Results

### 3.1. Time‐Dependent Persistence and Distribution of GFP‐Transduced hESC‐MSCs Following SC and IVT Injections

**Figure 2:**
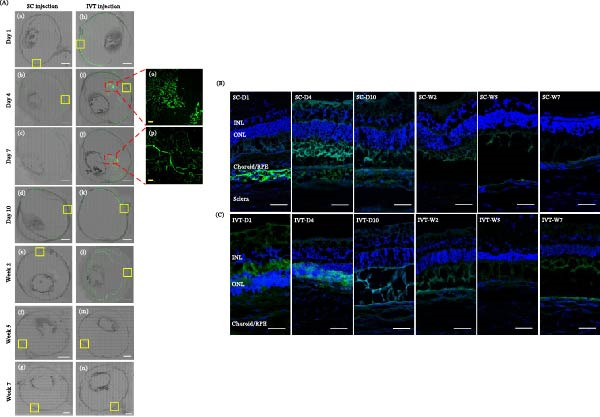
Analysis of GFP fluorescence signal persistence and distribution over time after intravitreal (IVT) and suprachoroidal (SC) injections. (A) Merged confocal images of GFP fluorescence in whole sagittal sections depict the distribution of GFP fluorescence signals following SC (a–g) and IVT (h–n) injections. IVT injections resulted in GFP fluorescence signals concentrated in the vitreous body for up to 7 days, as shown in magnified images (o, p) of (i, j). White scale bar = 2 mm, yellow scale bar = 100 µm. (B), (C) High‐magnification confocal images of the highlighted yellow‐boxed areas in (A) show GFP fluorescence signals and DAPI‐stained nuclei following SC injections at D1, D4, D10, W2, W5, and W7. IVT injections were examined at D1, D4, D7, W2, W5, and W7. This study analyzed one sample per time point, which limited direct comparisons. Scale bar = 50 µm.

SC injections showed detectable GFP fluorescence signals near the injection site on Day 1 (Figure 2Aa), with fluorescence extending toward the posterior segment by Day 4 (Figure 2Ab). The signals were visible for up to 2 weeks (Figure 2Ac–e), declined to near‐background levels at Week 5 (Figure 2Af), and were undetectable at Week 7 (Figure 2Ag). In contrast, IVT injections showed robust GFP fluorescence signals from Day 1 (Figure 2Ah), with prominent intravitreal (IVT) fluorescence aggregates apparent by Day 4 that were observed up to 1 week (Figure 2Ai, j). Signals disseminated toward the posterior region were gradually attenuated and became faint at Week 2 (Figure 2Ak, l). At Weeks 5 and 7, the signal pattern was comparable to that observed in the SC group (Figure 2Am, n).

Regarding distribution, GFP signals following SC injections were initially detected between the RPE/choroid and sclera on Day 1, with signals observed adjacent to the ONL by Day 4. Signal intensity declined by Day 10, merging with background autofluorescence by Week 2 and completely disappearing by Week 7 (Figure 2B). In the IVT injections, GFP signals were initially concentrated between the INL and ONL on Day 1, with flourescence signal distribution observed near the INL–ONL interface by Day 4. Signal intensity significantly decreased by Day 10; from Week 2 onward, the pattern became comparable to that of the SC injections (Figure 2C).

### 3.2. Retinal Function Analysis of SC‐Delivered hESC‐MSCs and PBS

**Figure 3:**
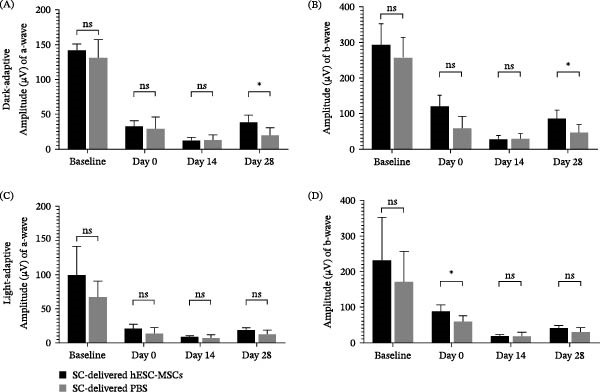
Electroretinogram (ERG) analysis of retinal responses under dark‐ and light‐adapted conditions following SC‐delivered hESC‐MSCs and PBS (*n* = 7 eyes per group). (A, B) Dark‐adapted a and b‐wave amplitudes. (C, D) Light‐adapted a‐ and b‐wave amplitudes. ERG recordings were obtained at baseline (pre‐RD induction), on Day 0 (24 hr after RD induction, immediately before hESC‐MSC/PBS injections), with follow‐up recordings on Day 14 and Day 28 post‐injection. Enucleation was performed on Day 28. Statistical significance was assessed using two‐way ANOVA with repeated measures. Data are presented as mean ± SEM.  ^∗^ *p* < 0.05, ns, not significant.

In the dark‐adapted condition, there were no significant between‐group differences in the a‐ and b‐wave amplitudes at baseline (pre‐RD induction) and Day 0 (24 hr after RD induction; immediately before hESC‐MSCs/PBS injections), indicating comparable initial conditions. Both amplitudes were significantly reduced on Day 0 compared with baseline (Figure 3A, B). By Day 14, neither amplitude showed improvement, suggesting no early therapeutic effect. By Day 28, the SC‐delivered hESC‐MSCs group showed a significant increase in the a‐wave (*p* = 0.039; hESC‐MSCs: 37.63 ± 7.02 vs. PBS: 19.58 ± 7.02) and b‐wave amplitudes (*p* = 0.034; hESC‐MSCs: 86.45 ± 14.74 vs. PBS: 46.51 ± 14.74) compared with the SC‐delivered PBS group.

In the light‐adapted condition, there were no significant between‐group differences at baseline. A significant between‐group difference was observed only in the b‐wave amplitudes on Day 0, whereas no significant differences were detected on Days 14 or 28 (Figure 3C, D).

### 3.3. ONL and INL Thickness Analysis of SC‐Delivered hESC‐MSCs and PBS

**Figure 4:**
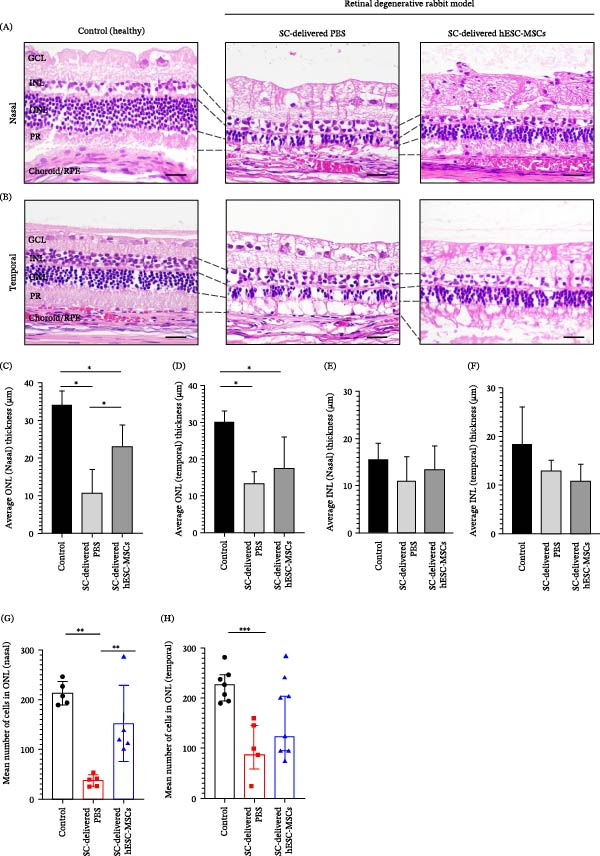
Analysis of outer nuclear layer (ONL) and inner nuclear layer (INL) thickness and cell count in eyes enucleated at Day 28 (post‐injection). (A, B). Hematoxylin and eosin (H&E)‐stained images of retinal sections from the control (healthy) (*n* = 5), SC‐delivered PBS group (*n* = 5), and SC‐delivered hESC‐MSCs group (*n* = 5) in the nasal (A) and temporal (B) regions. (C, D). ONL thickness in the nasal (C) and temporal (D) regions. (E, F). INL thickness in the nasal (E) and temporal (F) regions. (G, H). Mean number of cells in ONL of the nasal (G) and temporal (H) regions. Thickness and cell number were obtained 2–2.5 mm from ONH. Statistical analysis was performed using one‐way ANOVA and the Mann–Whitney *U* test.  ^∗^ *p* < 0.05 was considered statistically significant. Scale bar = 10 µm.

Compared with the control, SC‐delivered hESC‐MSCs and PBS‐groups showed a significant change in ONL thickness by Day 28; contrastingly, there was no significant change in INL thickness (Figure 4A, B). In the nasal region, the SC‐delivered hESC‐MSCs group exhibited a preservation effect, with the reduction in ONL thickness being smaller by 32.4% compared to the control (*p* = 0.0053, 23.08 µm ± 3.429 vs. 34.19 µm ± 3.249). Contrastingly, the SC‐delivered PBS group exhibited a significant reduction in ONL thickness compared with the control (*p* < 0.0001, 10.58 µm ± 3.429 vs. 34.19 µm ± 3.429) (Figure 4C, D).

A similar trend was observed in the temporal region, with the SC‐delivered hESC‐MSCs group demonstrating partial preservation of ONL thickness; however, the preservation effect was less pronounced than that in the nasal region. Specifically, the SC‐delivered hESC‐MSCs group had significantly lower ONL thickness than the control (*p* = 0.0019, 17.39 µm ± 3.523 vs. 30.09 µm ± 3.523); however, it was slightly elevated compared to that in the SC‐delivered PBS group, although this difference was not statistically significant. The SC‐delivered PBS group exhibited significantly reduced ONL thickness compared to the control (*p* = 0.0005, 13.38 µm ± 3.523 vs. 30.09 µm ± 3.523) (Figure 4E, F). Contrastingly, there were no significant between‐group differences in the INL thickness within nasal or temporal regions (Figure 4E, F).

Cell counting analysis revealed changes that were consistent with the thickness analysis. In the nasal region, the SC‐delivered hESC‐MSCs group showed a 42.9% reduction in cell density compared to the control, however it did not show statistically significant; the SC‐delivered PBS group showed a significant reduction compared to the control group (*p* = 0.0079, 39 vs. 207). The SC‐delivered hESC‐MSCs group had a higher cell density than the SC‐delivered PBS group (*p* = 0.0079, 120 vs. 39) (Figure 4G).

In the temporal region, the SC‐delivered PBS group showed a significantly reduced ONL cell number compared to the control (*p* = 0.0006, 87 vs. 227). The hESC‐MSCs group exhibited a slight increase in cell number relative to the SC‐delivered PBS group; however, this difference was not statistically significant (Figure 4H).

### 3.4. Analysis of Biological Markers of SC‐Delivered hESC‐MSCs and PBS

**Figure 5:**
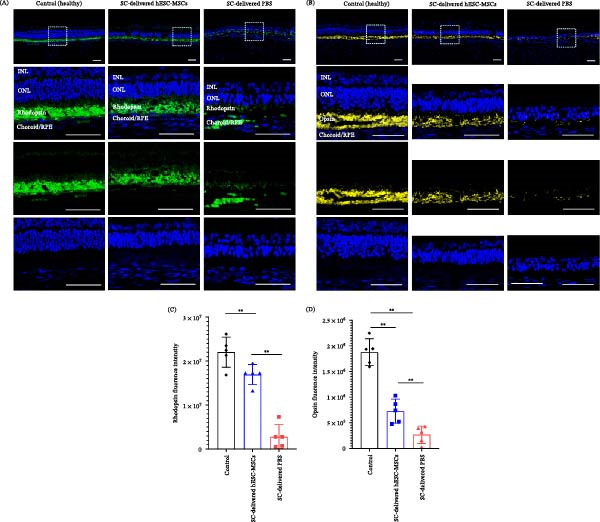
Rhodopsin and opsin expression levels were analyzed via immunofluorescence staining and quantified by intensity measurements from Day 28 enucleated eyes (post‐injection). (A, B) Visualization of rhodopsin and opsin expression using confocal microscopy. (C, D) Quantification of the fluorescence intensity of rhodopsin and opsin. Intensity measurements were performed in the same region where ONL and INL thickness were assessed (2–2.5 mm from the ONH) using 20× magnification. Intensity values were calculated as sum intensity. Statistical analysis was performed using the Mann–Whitney *U* test. Data are presented as the median with interquartile range (IQR) (*n* = 5 per group, same groups as those used for ONL and INL measurements).  ^∗^ *p* < 0.05 was considered statistically significant. Scale bar = 50 μm.

Immunofluorescence analysis revealed significant changes in rhodopsin (green) and opsin (yellow) signals in the SC‐delivered hESC‐MSC‐ and PBS‐groups relative to the control. Specifically, the SC‐delivered hESC‐MSCs group showed more intense and widespread fluorescence, especially in rhodopsin expression, while the SC‐delivered PBS group exhibited markedly diminished signals for both markers (Figure 5A, B). Fluorescence intensity quantification confirmed significantly greater rhodopsin signal intensity in the SC‐delivered hESC‐MSCs group than in the SC‐delivered PBS group (*p* = 0.007, 2.7 × 10^6^ vs. 1.7 × 10^6^, Figure 5C). Similarly, the opsin signal intensity was significantly higher in the SC‐delivered hESC‐MSCs group than in the SC‐delivered PBS group (*p* = 0.007, 7.4 × 10^5^ vs. 3.2 × 10^5^, Figure 5D); however, its signal remained weaker than that of rhodopsin. Contrastingly, both markers exhibited markedly reduced signal intensity in the SC‐delivered PBS group than in the control (*p* = 0.007).

### 3.5. Intraocular Inflammation Following SC or IVT Injections

**Figure 6:**
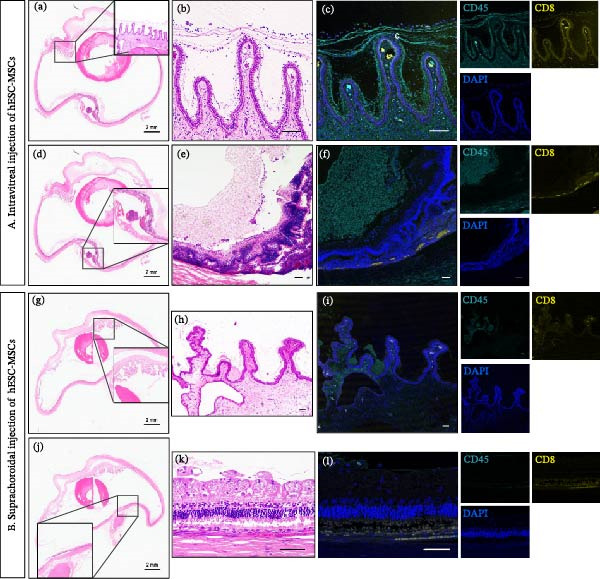
Histological and immunofluorescence analyses showing higher inflammation levels in eyes enucleated at Day 14 (post‐injection) with IVT injections than with SC injections. (A) (a) and (d) are whole‐eye scans following IVT injection (*n* = 3). (b) and (e) are magnifications of (a) and (d), respectively. There was significant inflammation in both anterior and posterior segments (c, f). Immunofluorescence staining shows CD45 (ocean blue) and CD8 (yellow) expression in (b, e). (B) (g) and (j) are whole‐eye scans following SC injection (*n* = 3). (h) and (k) are magnifications of (g) and (j), respectively. There was no inflammation in the anterior and posterior regions, with minimal CD45 (blue) and CD8 (yellow) staining in (h, k). Scale bar = 50 µm.

There were no visible inflammatory signs on the anterior segment, including hemorrhage or redness, by Day 14. However, histopathological analysis revealed significant intraocular inflammation in all IVT‐injected eyes (3/3), one SC‐injected eye (1/3), and no untreated RD‐induced eyes (0/2). Inflammatory responses in IVT‐injected eyes were localized around the inner epithelial layer of the ciliary body, the lens surface, and the posterior segment proximal to the inner limiting membrane (Figure 6Ab, c). Immunofluorescent staining with CD45 and CD8 further confirmed the presence of inflammatory markers in all IVT‐injected eyes. Contrastingly, SC‐injected eyes exhibited minimal staining, which indicated a negligible inflammatory response (1/3) (Figure 6Bh–l).

## 4. Discussion

This study showed that SC hESC‐MSC injection restored retinal function and structure from sodium iodate‐induced RD. Moreover, inflammation was negligible with the SC hESC‐MSC injection, whereas a pronounced inflammatory response was observed at Day 14 following IVT injection. Therefore, further efficacy evaluation was not pursued.

Worldwide, RD is a leading cause of irreversible blindness, and its annual prevalence continues to increase [35, 36]. However, its therapeutic options remain limited. Despite increasing interest in MSC‐based therapies in ophthalmology [13, 14], MSC delivery to the retina is challenging given the complexity of the eye structure. Photoreceptors in the retina are primarily involved in cellular stress, dysfunction, and death observed during RD. It is difficult to target photoreceptors with MSCs through IVT injection since they are located in the outermost retinal layer [37]. Although subretinal delivery is an anatomically relevant route for this, it necessitates complex surgical procedures and carries inherent risks [3, 4, 6]. Accordingly, the SCS has been explored as an alternative route. However, its clinical utility is hindered by its use of invasive techniques that offer no clear advantage over subretinal delivery with respect to efficacy or feasibility [6, 15, 38].

Recent clinical studies have investigated the utility of SC delivery in retinal therapies, including AAV gene therapy, nonviral gene‐based therapy, and other small‐molecule treatments [39]. However, SC delivery in stem cell therapy is limited by concerns regarding ocular inflammation, rapid clearance, and the absence of comprehensive reports [9, 20]. Therefore, we evaluated the therapeutic potential for SC delivery of hESC‐MSCs in a rabbit RD model. The SCS was accessed using custom‐designed 3D‐printed caps, which provided a straightforward, efficient, and safer alternative to conventional methods, enabling precise and minimally invasive cell delivery [15, 40].

Notably, our findings suggest that SC delivery effectively addresses the challenge of targeting the photoreceptor layer in RD. By enabling targeted delivery to the posterior segment without entering the vitreous cavity, SC delivery may overcome the anatomical limitations impeding IVT administration. This approach may reduce the risk of complications associated with prolonged IVT residence [4, 6, 24, 28]. Furthermore, SC injections have been shown to allow rapid clearance compared with IVT injections, especially for small molecules. The clearance rates in SC injections are affected by molecular size, weight, and solubility [15, 23, 25, 26, 41]. In the present study, GFP fluorescence signals after SC injection were detectable by visual inspection up to Week 2, the duration was not shorter than that observed after IVT injections. Alu‐sx PCR analysis detected hESC‐MSCs’ genomic DNA in the SC injection within 2 weeks post‐transplantation, with none detected thereafter (Figure S5). However, this analysis is limited by the use of a single sample per time point. Notably, following IVT administration, initial diffusion within the vitreous cavity may disperse cells away from the retinal layers, whereas SC injection may localize cells closer to the photoreceptors, which is presumed to provide a more favorable proximity for paracrine signaling.

Additionally, we assessed the therapeutic efficacy of hESC‐MSCs delivered using SC injections using ERG testing, morphological analysis of the ONL and INL, and analysis of photoreceptor‐specific biomarkers such as opsin and rhodopsin [42, 43]. Notably, there was a significant difference under dark‐adapted, but not light‐adapted conditions between hESC‐MSC‐ and PBS‐treated eyes. Additionally, there was a significant increase in ONL and cell number, but not INL thickness, in the hESC‐MSC‐treated group, which is consistent with previous findings [33]. Consistently, the hESC‐MSC‐treated group showed stronger expression of rhodopsin and opsin, with the former showing more pronounced expression. Taken together, these findings illustrate that hESC‐MSC treatment conferred a substantial preservation effect on both retinal architecture and functionality. Additionally, given the more pronounced upregulation of rhodopsin expression, hESC‐MSC therapy may predominantly influence rod photoreceptor function. This may explain the observed significant improvements in the dark‐adapted, but not light‐adapted, ERG responses. These observations imply that hESC‐MSC treatment may initially target rod photoreceptors; further, with extended follow‐up or additional administrations, the functional recovery may then extend to cone photoreceptors. Notably, although hESC‐MSC function may decline with increasing passage number, we observed significant structural and functional benefits using GMP‐manufactured P12 cells that met predefined release criteria; however, passage‐related effects cannot be fully excluded. In addition, dose optimization and a formal dose–response evaluation were beyond the scope of this study and warrant further investigation.

In our study, the SC route was associated with minimal ocular inflammation compared with the IVT route, and further studies are warranted to elucidate the underlying mechanisms. IVT‐administered hESC‐MSCs may distribute in proximity to the INL, a region where microglial cells are predominantly found. MSC‐associated microglial activation may trigger an inflammatory cascade, which contributes to retinal disruption [3, 44–48]. Notably, the SC route bypasses the vitreous cavity to directly deliver cells to the posterior segment of the eye and minimizes contact with the INL, thereby potentially reducing microglial activation and inflammatory response relative to IVT injections.

## 5. Conclusion

SC delivery could provide a viable alternative to nontargeted IVT delivery in RD, addressing inflammation and overcoming key limitations of current approaches.

## Author Contributions

**Hyun Hee Seo**: data curation, formal analysis, investigation, methodology, validation, visualization, writing – original draft, writing – review and editing. **Eun Ju Lee**: resources, writing – review and editing. **Yoon Young Kim**: resource, supervision. **Seung-Yup Ku**: resources, writing – review and editing. **Chang Ki Yoon**: conceptualization, formal analysis, funding acquisition, investigation, supervision, writing – review and editing.

## Funding

This work was supported by a grant from the Korea Health Technology R&D Project through the Korea Health Industry Development Institute (KHIDI), funded by the Ministry of Health & Welfare, Republic of Korea (Grant HI14C1277).

## Disclosure

A version of this manuscript has been previously deposited as a preprint on ResearchGate (DOI: 10.2139/ssrn.5211350). The authors alone are responsible for the content and writing of the article.

## Conflicts of Interest

The authors declare no conflicts of interest.

## Supporting Information

Additional supporting information can be found online in the Supporting Information section.

## Supporting information


**Supporting Information** Figure S1: Verification of the feasibility of suprachoroidal injections using 3D‐printed needle caps. Red FluoSphere microspheres (150 µL) were injected using customized 3D‐printed needle caps and were detected between the RPE/choroid and sclera. A representative image was created by merging over 150 confocal images; INL, inner nuclear layer; ONL, outer nuclear layer. Scale bar = 100 µm. Figure S2: Identification of human embryonic stem cell‐derived mesenchymal stem cells (hESC‐MSCs) and GFP‐labeled hESC‐MSCs. (A) Light microscopy images show that GFP‐transduced hESC‐MSCs remained stable and proliferated for up to 24 h. (B) Strong GFP expression is evident under brightfield microscopy (left) and was confirmed by fluorescence microscopy (right). Figure S3: Flow cytometric immunophenotyping of GFP‐transduced hESC‐MSCs. Representative gating strategy (top) and corresponding histograms (bottom) demonstrate the sequential gating process, confirming negative expression of the hematopoietic marker CD45 and positive expression of canonical MSC surface markers (CD73, CD90, and CD105) following GFP transduction. Figure S4: Electroretinography (ERG) responses after NaIO3 injection (15 or 20 mg/kg) in rabbits. (A, B) Scotopic a‐wave, (C, D) Scotopic b‐wave, (E, F) Photopic a‐wave, and (G, H) Photopic b‐wave amplitudes were measured at baseline (NaIO3 injection) and on Days 1, 3, and 7 after NaIO3 injection across flash intensities (colors denote time points). Data are shown as mean values (1 rabbit per dose; nn = 2 eyes per dose); no inferential statistics were performed. Figure S5: TPA25 (human Alu region) PCR gel for detection of human‐derived signals. M, 1 kb DNA ladder; Lane 1, hESC‐MSC (+); Lane 2, noninjected healthy rabbit eye (−); Lanes 3–8, IVT/SC injections at Day 7, Day 14, and Week 5. The target bands (~400–500 bp) are visible in lanes 1, 4, and 6, while larger bands in lane 2 were nonspecific.

## Data Availability

The data that support the findings of this study are available from the corresponding author upon reasonable request.
