# Supplementary material for: Therapeutic Evaluation of the Suprachoroidal Space for Stem Cell Delivery in Retinal Degeneration
Source: Stem Cells Int. 2026 Apr 27;2026:1374159. doi: 10.1155/sci/1374159 (PMC13121866; doi:10.1155/sci/1374159)
Supplement: Supplementary file 1 — Supporting Information Figure S1: Verification of the feasibility of suprachoroidal injections using 3D‐printed needle caps. Red FluoSphere microspheres (150 µL) were injected using customized 3D‐printed needle caps and were detected between the RPE/choroid and sclera. A representative image was created by merging over 150 confocal images; INL, inner nuclear layer; ONL, outer nuclear layer. Scale bar = 100 µm. Figure S2: Identification of human embryonic stem cell‐derived mesenchymal stem cells (hESC‐MSCs) and GFP‐labeled hESC‐MSCs. (A) Light microscopy images show that GFP‐transduced hESC‐MSCs remained stable and proliferated for up to 24 h. (B) Strong GFP expression is evident under brightfield microscopy (left) and was confirmed by fluorescence microscopy (right). Figure S3: Flow cytometric immunophenotyping of GFP‐transduced hESC‐MSCs. Representative gating strategy (top) and corresponding histograms (bottom) demonstrate the sequential gating process, confirming negative expression of the hematopoietic marker CD45 and positive expression of canonical MSC surface markers (CD73, CD90, and CD105) following GFP transduction. Figure S4: Electroretinography (ERG) responses after NaIO3 injection (15 or 20 mg/kg) in rabbits. (A, B) Scotopic a‐wave, (C, D) Scotopic b‐wave, (E, F) Photopic a‐wave, and (G, H) Photopic b‐wave amplitudes were measured at baseline (NaIO3 injection) and on Days 1, 3, and 7 after NaIO3 injection across flash intensities (colors denote time points). Data are shown as mean values (1 rabbit per dose; nn = 2 eyes per dose); no inferential statistics were performed. Figure S5: TPA25 (human Alu region) PCR gel for detection of human‐derived signals. M, 1 kb DNA ladder; Lane 1, hESC‐MSC (+); Lane 2, noninjected healthy rabbit eye (−); Lanes 3–8, IVT/SC injections at Day 7, Day 14, and Week 5. The target bands (~400–500 bp) are visible in lanes 1, 4, and 6, while larger bands in lane 2 were nonspecific. [file SCI-2026-1374159-s001.pdf]

Supplementary Figure S1. Verification of the feasibility of suprachoroidal injections using 3D-printed needle caps. Red FluoSphere microspheres (150 μL) were injected using customized 3D-printed needle caps and were detected between the RPE/choroid and sclera. A representative image was created by merging over 150 confocal images; INL, inner nuclear layer; ONL, outer nuclear layer. Scale bar = 100 μm.

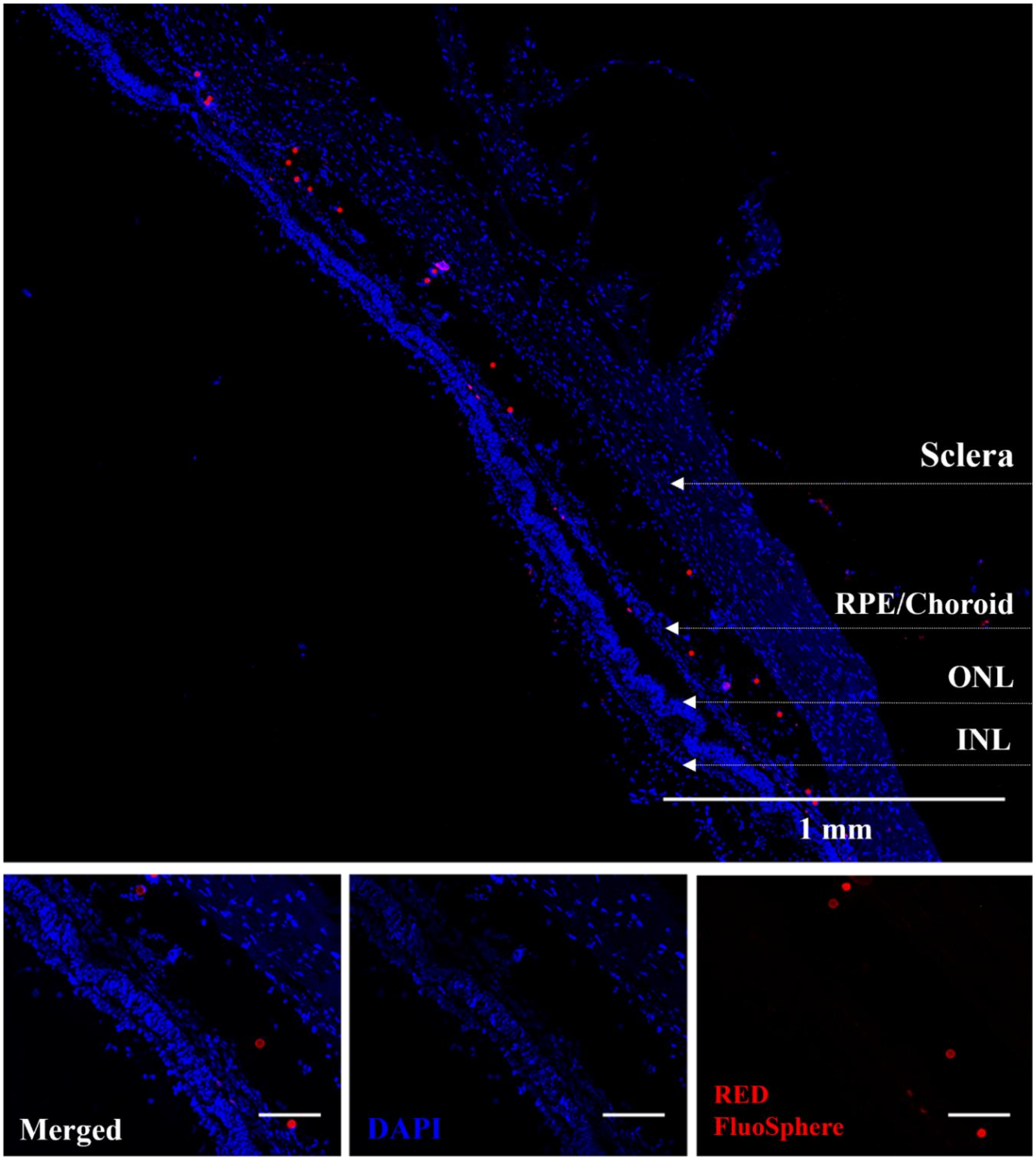

Supplementary Figure S2. Identification of human embryonic stem cell-derived mesenchymal stem cells (hESC-MSCs) and GFP-labeled hESC-MSCs. **(A)** Light microscopy images show that GFP-transduced hESC-MSCs remained stable and proliferated up to 24 h. **(B)** Strong GFP expression is evident under brightfield microscopy (left) and was confirmed by fluorescence microscopy (right).

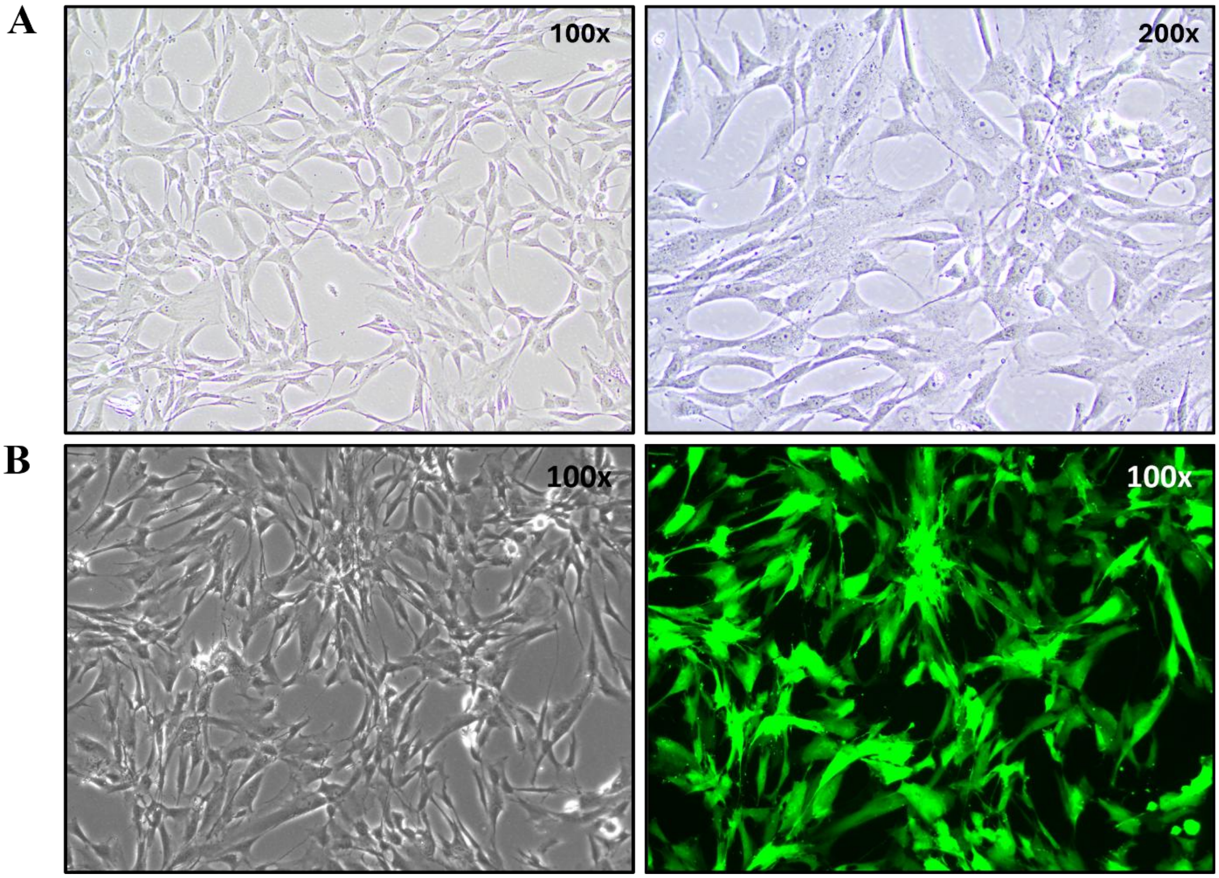

Supplementary Figure S3. Flow cytometric immunophenotyping of GFP-transduced hESC-MSCs. Representative gating strategy (top) and corresponding histograms (bottom) demonstrate the sequential gating process, confirming negative expression of the hematopoietic marker CD45 and positive expression of canonical MSC surface markers (CD73, CD90, and CD105) following GFP transduction.

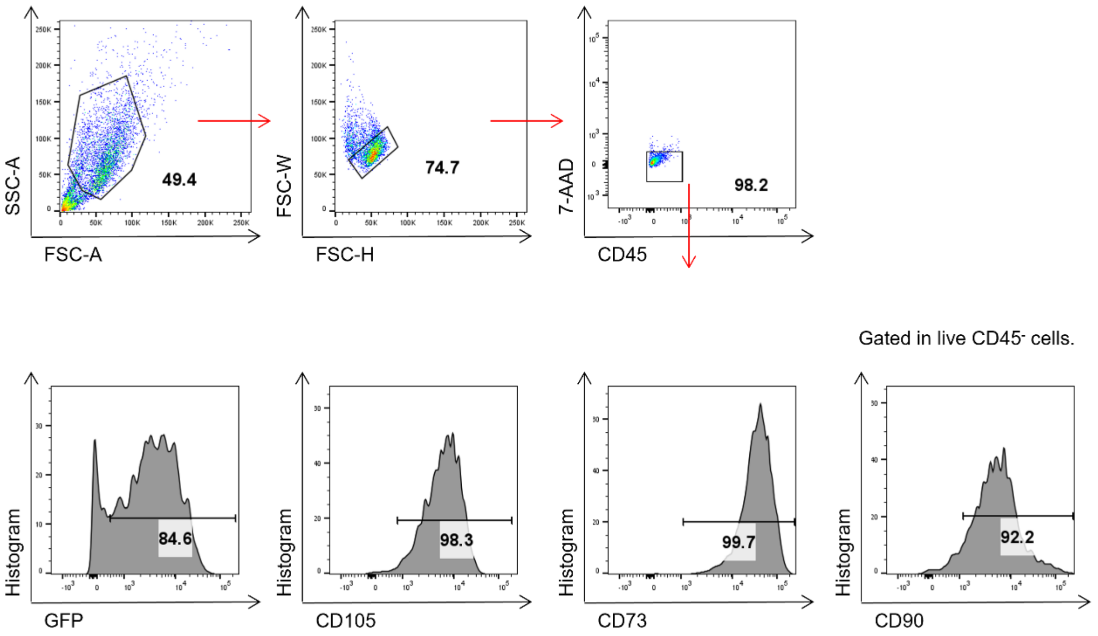

Supplementary Figure S4. Electroretinography (ERG) responses after $NaIO_3$ injection (15 or 20 mg/kg) in rabbits. **(A, B)** Scotopic a-wave, **(C, D)** Scotopic b-wave, **(E, F)** Photopic a-wave, and **(G, H)** Photopic b-wave amplitudes were measured at baseline ($NaIO_3$ injection) and on Day 1, 3, and 7 after $NaIO_3$ injection across flash intensities (colors denote time points). Data are shown as mean values (1 rabbit per dose; n = 2 eyes per dose); no inferential statistics were performed.

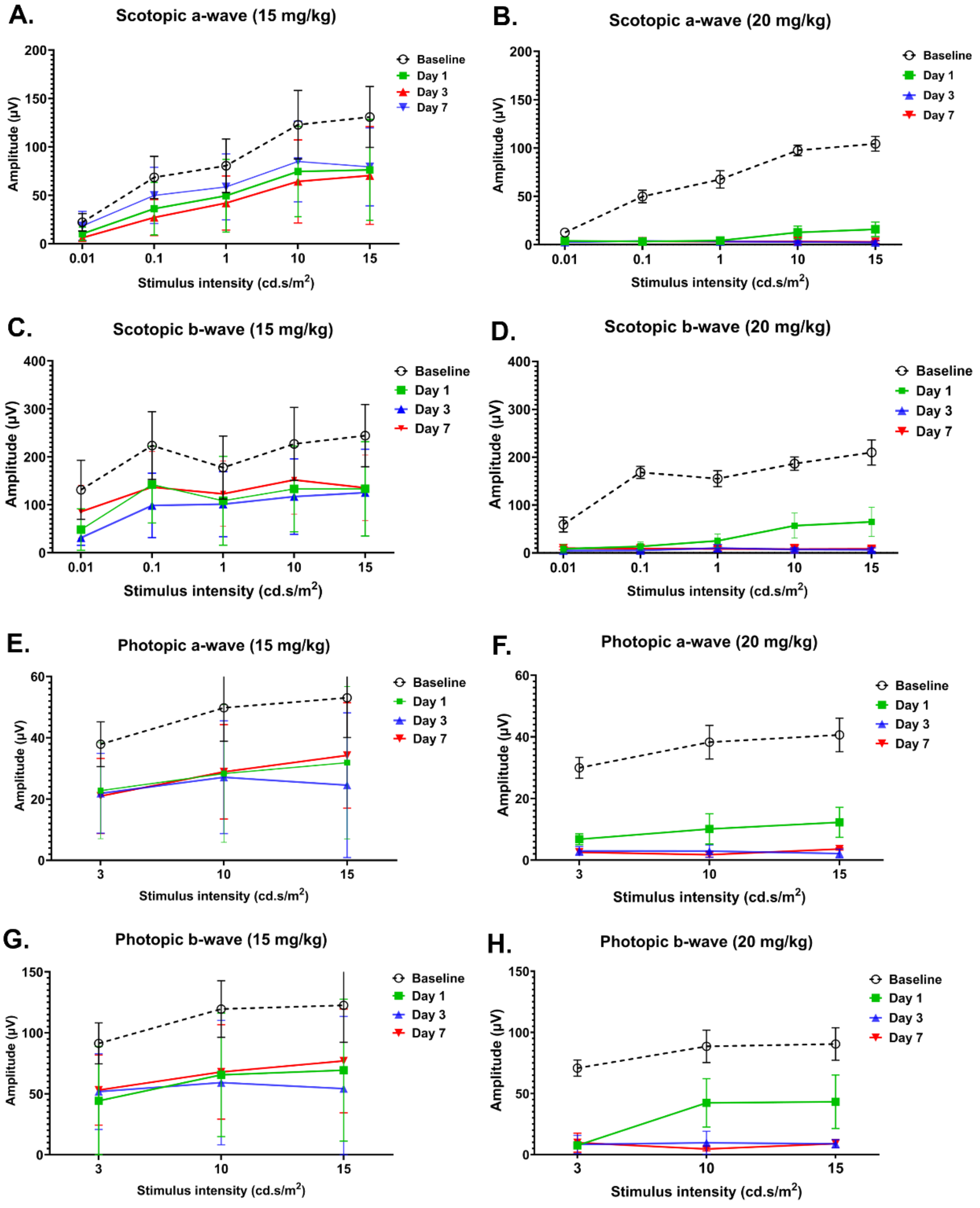

Supplementary Figure S5. TPA25 (human Alu region) PCR gel for detection of human-derived signals. M, 1 kb DNA ladder; Lane 1, hESC-MSC (+); Lane 2, non-injected healthy rabbit eye (−); Lanes 3–8, IVT/SC injections at Day 7, Day 14, and Week 5. The target bands (~400–500 bp) are visible in lanes 1, 4, and 6, while larger bands in lane 2 were non-specific.

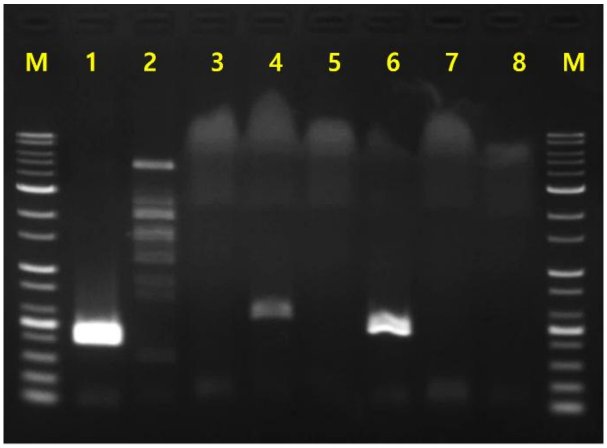

M 1kb DNA Ladder
1. hESC-MSC
2. Negative Control: Non-injected healthy rabbit eye
3. IVT injection at Day 7
4. SC injection at Day 7
5. IV injection at Day 14
6. SC injection at Day 14
7. IV injection at Week 5
8. SC injection at Week 5
